# The development and implementation of a low-cost mechanical ventilator in a low-middle-income country during the COVID-19 pandemic: The Unisabana-HERONS

**DOI:** 10.1016/j.heliyon.2024.e30671

**Published:** 2024-05-05

**Authors:** Luis Fernando Giraldo-Cadavid, Julian Echeverry, Fabio Varón-Vega, Alirio Bastidas, Andrés Ramírez-Jaime, Andrés Felipe Cardona, Cristian Joao Lopez Vega, Cristian C. Serrano-Mayorca, Diana Garay, Diego Nicolás Rincón, Henry Oliveros, Iván Arturo Ramírez, Esteban Garcia-Gallo, Valeria A. Enciso-Prieto, Elsa D. Ibáñez-Prada, Juan Carlos Camelo, Laura Cucunubo, Lina Buitrago, Luis Alfredo Paipa, Luis Carlos Longas, Luis Mauricio Agudelo-Otálora, Nestor Fernando Porras Diaz, Rolando Roncancio Rachid, Rubén Darío Henao I, Santiago Pedraza, Luis Felipe Reyes

**Affiliations:** a Universidad de La Sabana, Chía, Colombia; b Fundación Neumológica Colombiana, Bogotá, Colombia; c Universidad de Navarra, Pamplona, Spain; d Universidad de Los Andes, Bogotá, Colombia; e Fundación Clínica Shaio, Bogotá, Colombia; f Fundación Cardioinfantil Instituto de Cardiología, Bogotá, Colombia; g Universidad Nacional de Colombia, Bogotá, Colombia; h Clínica Universidad de La Sabana, Chía, Colombia; i Pandemic Sciences Institute, University of Oxford, Oxford, United Kingdom

**Keywords:** COVID-19, Invasive mechanical ventilator, Respiratory failure

## Abstract

**Background:**

The COVID-19 pandemic in Latin America generated the need to develop low-cost, fast-manufacturing mechanical ventilators. The Universidad de La Sabana and the Fundacion Neumologica Colombiana designed and manufactured the Unisabana-HERONS (USH) ventilator. Here, we present the preclinical and clinical study results to evaluate its effectiveness and safety characteristics in an animal model (Y*orkshire Sow)* and five patients with acute respiratory failure receiving mechanical ventilatory support for 24 h.

**Methods:**

The effectiveness and safety outcomes included maintaining arterial blood gases and pulse oximetry saturation (SpO2), respiratory pressures and volumes (during continuous monitoring) in the range of ARDS and lung-protective strategy goals, and the occurrence of barotrauma. A significance level of 0.05 was used for statistical tests. This clinical trial was registered on Clinicaltrials.gov (NCT04497623) and approved by the ethics committee.

**Results:**

Among patients treated with the Unisabana-HERONS, the most frequent causes of acute respiratory failure were pneumonia in 3/5 (60 %) and ARDS in 2/5 (40 %). During the treatment, the ventilatory parameters related to lung protection protocols were kept within the safety range, and vital signs and blood gas were stable. The percentage of time that the respiratory pressures or volumes were out of safety range were plateau pressure >30 cm H2O: 0.00 %; driving pressure >15 cm H2O: 0.06 %; mechanical power >15 J/min: 0.00 %; and Tidal volume >8 mL/kg: 0.00 %. There were no adverse events related to the ventilator. The usability questionnaire retrieved a median score for all items between 9 and 10 (best score: 10), indicating great ease of use.

**Conclusion:**

The Unisabana-HERONS ventilator effectively provided adequate gas exchange and maintained the ventilatory parameters in the range of lung protection strategies in humans and an animal model. Furthermore, it is straightforward to use and is a low-cost medical device.

## 1 Introduction

In December 2019, a new highly contagious Severe Acute Respiratory Syndrome Coronavirus- 2 (SARS-CoV-2) was identified in China, responsible for more than 800 million infections and up to 7 million deaths in the last three years (https://covid19.who.int/) [1]. The presentation of coronavirus disease 2019 (COVID-19) varies from asymptomatic to systemic disease [2,3]. Around 54 % of patients who receive in-hospital management develop respiratory failure, and 31 % of those with acute respiratory distress syndrome (ARDS) require intensive care unit (ICU) admission to receive advanced ventilatory support [4,5]. Due to the high prevalence of ARDS, the stockpiling and availability of ventilators became a public health concern, especially in low and middle-income countries (LMIC) [6,7]. Each mechanical ventilator costs around 30.000 USD, and on top of that, the manufacturing of mechanical ventilators is centralized in developed countries; thus, these countries prioritized ventilators for their local consumers rather than exporting them [8,9].

Since the obtention of conventional ventilators was not feasible, and some countries could not access them due to the unavailability of pressurized gas and other socioeconomic barriers, numerous low-cost ventilation devices were developed [10]. Moreover, since the beginning of the pandemic, different governments worldwide have predicted an imminent shortage of ventilators due to the saturation of the supply chain during the pandemic and competitive priorities [11]. Some countries even launched initiatives to develop locally produced ventilators at lower prices and with the capacity to scale up their production rapidly. Therefore, several countries, including the Federal Drug Administration (FDA) in the United States, adjusted their regulatory processes to evaluate and approve the newly developed ventilators, and different initiatives to produce new low-cost ventilators were launched [10,11]. For instance, the Fast-AssembLy COVID-Nineteen (FALCON) ventilator prototype showed promising results on artificial lung models [10]. Moreover, OxVent accomplished the specifications provided by the Medicines and Healthcare Products Regulatory Agency (MHRA) for rapidly manufactured ventilator systems (RMVS) in artificial lungs and animal models [11]. It is the same as the ResUHUrge ventilator developed in Spain [12]. However, these were non-certified, and most remained as provisional prototypes; therefore, the studies did not proceed to the following stages in humans. Hence, LMICs were still depending on the supply chain from wealthy countries, and consequently, they focused on expanding the ICU capacity [13,14]. Therefore, it is still unclear if a country with limited resources, such as Colombia, could design, produce, and test in both animals and humans a low-cost ventilator that could be safely used in patients during a pandemic to overcome the ventilator shortage. Therefore, this study was designed to bridge this gap in the literature.

In this study, we developed a low-cost mechanical ventilator, the Unisabana-HERONS, as an alternative to regular ventilator devices unavailability in LMICs such as Colombia. To ensure the safety and effectiveness of this ventilator, it was tested in animal and human models.

## 2 Materials and methods

To create and develop the Unisabana-HERONS ventilator, we divided the process into three stages: phase 1, design and production; phase 2, the preclinical study testing using a lung simulator and animal experiments; and phase 3, testing the ability of the ventilator to provide safe oxygenation in humans. The functional and pig (*Yorkshire Sow)* model experiments were executed at Fundación Neumológica Colombiana (CEIVEM) and Fundación Instituto De Simulación Médica (INSIMED), respectively. The study was approved by the Institutional Animal Care and Use Committee (cicuas_20_086) following the Colombian National Guidelines according to Colombian guidelines stated in Law 84 of 1989 and Decree 8430 of 1993. The human studies were performed at the Clínica Universidad de La Sabana in Chía, Colombia, and the institutional ethics board committee approved all procedures (MED-328-2022) by the Comité de Ética en Investigación y Farmacología (Acta 09–20) (Fig. S1). A signed written consent by the participant patient or his legal/familiar representative was required to participate. The study was also approved by the Colombian Regulatory Agency for Drugs, food, and Medical Devices (INVIMA) and was registered on Clinicaltrials.gov (NCT04497623).

### 2.1 Phase 1: design and development of the Unisabana-HERONS

**Fig. 1:**
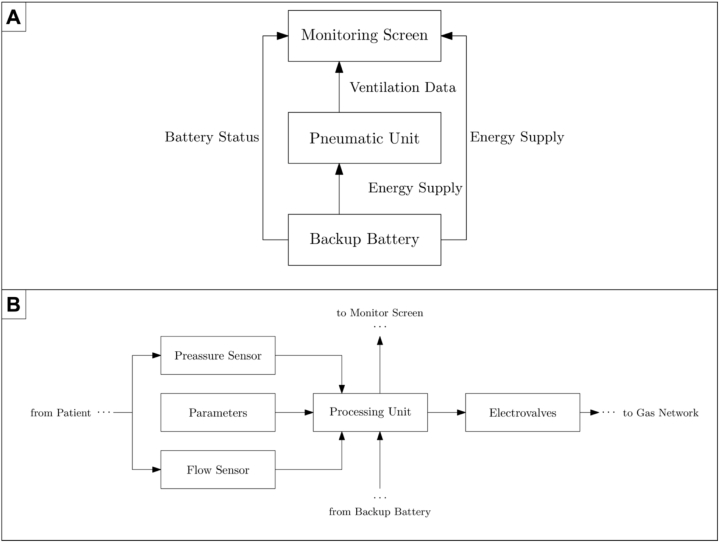
**Composition of the Unisabana-HERONS ventilator.** Three fundamental units are required: a monitoring screen, a pneumatic unit, and a backup battery.

Based on the International Organization for Standardization (ISO) criteria, the Unisabana-HERONS ventilator is an invasive mechanical ventilator that provides volume-controlled ventilation with continuous positive airway pressure (CPAP). It comprises three fundamental units: a monitoring screen, a pneumatic unit, and a backup battery (Fig. 1A and B). The pneumatic unit is responsible for ventilating the patient and collecting all the information about the airways; it has a system of electro valves that allow air or oxygen extraction from the installation's gas network or oxygen cylinders to ventilate the patients with a determinate FiO2. Its central processing unit has sensors that allow pressure measurement (using a membrane sensor) over the airways and the flow (utilizing a Venturi sensor) delivered to the patient. The volume calculations are made with numerical integration of the flow measurements.

Additionally, it performs practical calculations to ensure safe ventilation, such as conduction pressure, plateau pressure, mechanical power, and lung compliance. All the information can be retrieved at any time from internal memory. The ventilator works with an electrical outlet, has a backup battery unit that provides electrical power to the entire system, and works online; hence, optimal levels are guaranteed in grid-connected and off-grid operations.

### 2.2 Phase 2: preclinical studies

Functional tests of the Unisabana-HERONS ventilator were carried out in a lung simulator from the training and research center's mechanical ventilation of CEIVEM. The conditions of a healthy lung and a severe ARDS-affected lung were simulated. The positive end expiration pressure (PEEP) stability, the fraction of inspired oxygen (FiO2), and the concordance between the ventilator and the simulator for pressure flow and volume were verified. Also, the concordance between the programmed inspiration and the measured expiration ratios was evaluated. Additional information is presented in the only supplement.

Animal studies in a pig (*Y. Sow*) model were conducted in the INSIMED foundation. Pigs (*Y. Sow*) underwent sedation, neuromuscular blocking, and ventilator performance tests executed in healthy individuals and those with induced ARDS. The effect of the increase in PEEP and FiO2 on oxygen saturation (SaO2) and partial arterial oxygen tension (PaO2) was observed. The effectiveness of increasing PEEP to increment PaO2 and minute volume to decrease the partial arterial carbon dioxide tension (PaCO2) was validated. Regarding the tests in models with ARDS, they were induced with the bronchoalveolar lavage technique plus volume ventilator-induced lung injury caused by ventilation with volumes between 15 and 20 cc/kg. Bronchoalveolar lavage was done with normal saline solution at 30 cc/kg until achieving a PaO2/FiO2 ratio below 100, compliance below 20 mL/cmH2O, and B lines and white lung areas were observed in the chest ultrasound in upper and lower lobes.

### 2.3 Phase 3: safety studies in humans

#### 2.3.1 Study design and study population

To validate the safety and effectiveness of the Unisabana-HERONS in humans, a prospective cohort was recruited at Clínica Universidad de La Sabana. Eligibility criteria: patients who required UCI admission and were under mechanical ventilatory support on volume control mode with an expected duration greater than 24 h between July 1, 2020, and August 20, 2020, were included. Patients must have also fulfilled any of the following inclusion criteria [1]: Acute respiratory failure (PaO2/FiO2 <300) requiring mechanical ventilation [2], postoperative requiring ventilatory support [3], head trauma with the indication of mechanical ventilatory support [4], acute intoxication and respiratory depression with an indication of mechanical ventilatory supply.

Patients with the following conditions were excluded [1]: pregnancy [2], hypotension with mean arterial pressure (MAP) < 65 mmHg [3], PaO2/FiO2 <100 [4], brain edema or requirement of neurological protection [5], suspected intracranial hypertension [6], SOFA score >9 points [7], patients who were already receiving mechanical ventilation with a PEEP> 8 cmH2O, plateau pressure >30 cm H2O, or FiO2 >70 % [8], patients with confirmed or high clinical suspicion of COVID-19. Patients were considered obese if their body mass index was greater than 30. All the other clinical definitions are in the online supplement.

Regarding the training for professionals, sixteen people received online training before they used the ventilator. After testing, they must answer nine questions about the Unisabana-HERONS ventilator usability. Each question was scored on a numerical scale from 0 to 10, where 0 corresponded to the inability to configure a particular ventilator parameter and 10 to extremely easy to set.

#### 2.3.2 Monitored variables and measures

The data were recorded in a secure server hosted by the Universidad de La Sabana using an electronic case report form (CRF) in Redcap. The target ventilatory parameters for each patient were established according to the protective lung ventilation strategy [15], adjusting PaO2, PCO2, and oxygen saturation to Bogota's (altitude of 2600 m above sea level) normal values [16] (Table S13). Such ventilatory parameters were scheduled to be measured 30 min before the connection to the Unisabana-HERONS mechanical ventilator, then every 15 min for the first 4 h, and every hour from 4 to 24 h after the connection. Arterial blood gases were taken and registered every 30 min during the early 4 h, and in the next 20 h, they were only taken 12 and 24 h after connection. The scores of Acute Physiology and Chronic Health Disease Classification System II (APACHE) and Sepsis-related Organ Failure Assessment (SOFA) were calculated to stratify severity. Additionally, the registries of the vital signs from the monitors and the Unisabana-HERONS ventilator were retrieved.

### 2.4 Statistical analysis

Qualitative variables were described in frequencies and proportions. The quantitative variables were described with means and standard derivation (SD) or medians and interquartile ranges (IQR) according to their distribution. A significance level of 0.05 was used for statistical tests.

A binary cumulative sum analysis (CUSUM analysis) was performed to evaluate the frequency in which the ventilatory safety parameters were out of range. This method allows continuous monitoring of a production process, a professional's competence, or a device's performance to detect subtle deviations from a previously defined performance level [[17], [18], [19]]. It was considered an acceptable error when 10 % of the measurements were outside the safety ranges and an unacceptable error when 20 % or more were outside those ranges. It was defined as a type I error (a) of 0.1 and a type II error (b) of 0.1 according to the usual recommendations for this method [[17], [18], [19]] (see details in the S1 supplementary appendix).

*Sample size*: Detecting a difference ≥20 % in the mean values [20] of the outcomes (PaO2, SaO2, PaCO2, HCO3, PaFi, SaFi) implied a standardized effect size between 1 and 2 and assuming a coefficient of variation of these variables between 2 % and 9 % [21], a confidence of 95 % and a power of 80 %, this gives a sample size of 4–10 repeated measurements [22]. The sample size for the CUSUM binary analysis for judging if a variable under study remains in the established range (control chart for attributes) is at least 50 repeated measurements [23]. Therefore, the minimum sample size for this study was established in 50 repeated measurements of the outcome variables.

The following statistical packages were used for the statistical analysis: Microsoft Excel 2007, Stata version 16, and R 4.2.3. R Core Team (2023). R: A language and environment for statistical computing and MedCalc version 19.4.1.

## 3 Results

### 3.1 Preclinical phase

The ventilator was tested on two pigs (*Y. Sow*). Healthy pigs (*Y. Sow*) ventilated with the Unisabana-HERONS showed the PEEP and FiO2 effect in the oxygen saturation and PaO2; as the PEEP was increased from 7 to 15 HO2cm with a FiO2 of 100 %, the PO2 changed from 171 to 467 mmHg. Also, the PaCO2 decreased from 57.1 mmHg to 40.5 mmHg with an increase in the minute ventilation from 5.9 to 7.8 L/min. Then, we ventilated them once they developed ARDS and found that the rise of PEEP from 10 to 15 cmHO2 with an FIO2 of 100 % improved the oxygen saturation from 82 % to 91 % and the PaO2 from 53.2 to 71.6 mmHg. Additionally, monitoring the pressure plateau allowed the reduction of pulmonary volumes to the protection ranges and avoided adverse effects or ventilator-related adverse events shown in Table S14.

### 3.2 Patient's medical characteristics

A total of 5 patients were included; most were males (60 % [3/5]) with a median (IQR) age of 56.0 years (43.5–65.5) old. The most frequent diagnosis at admission was an acute hypoxemic respiratory failure (80 % [4/5]), followed by septic shock (60 % [3/5]), pneumonia (60 % [3/5]), and ARDS (40 % [2/5]). The most frequent comorbidity was systemic arterial hypertension (60 % [3/5]), while diabetes mellitus, hypothyroidism, solid neoplasm, obesity, and multiple trauma were only presented once in the cohort. Regarding severity, SOFA and APACHE scores had a median (IQR) of 5.0 (3.0–6.0) and 6.0 (4.0–9.5), respectively, at admission. All patients' characteristics are shown in Table 1.

**Table 1:** General characteristics of the cohort.

Characteristic	All Cohort, n = 5
Sex Males, n (%)	3.0 (60.0 %)
Age, median (IQR)	56.0 (43.5–65.5)
BMI, median (IQR)	24.2 (22.6–28.6)
**Diagnosis at Admission**	
Hypoxemic ventilatory failure, n (%)	4.0 (80.0 %)
Pneumonia, n (%)	3.0 (60.0 %)
Septic shock, n (%)	3.0 (60.0 %)
SDRA, n (%)	2.0 (40.0 %)
POP head and neck surgery, n (%)	1.0 (20.0 %)
Expansive neck hematoma, n (%)	1.0 (20.0 %)
**Comorbidities**	
High blood pressure, n (%)	3.0 (60.0 %)
Diabetes mellitus, n (%)	1.0 (20.0 %)
Hypothyroidism, n (%)	1.0 (20.0 %)
Solid neoplasm, n (%)	1.0 (20.0 %)
Obesity, n (%)	1.0 (20.0 %)
Multiple trauma, n (%)	1.0 (20.0 %)
SOFA (hour 0), median (IQR)	5.0 (3.0–6).0
SOFA (24th hour), median (IQR)	5.0 (3.0–6.0)
APACHE II (hour 0), median (IQR)	6.0 (4.0–9.5)
APACHE II (hour 24), median (IQR)	6.0 (4.0–9.0)
Abbreviations: BMI, body mass index; IQR: interquartile range (25th to 75th percentile); SOFA, Sequential Organ Failure Assessment; APACHE, Acute Physiology and Chronic Health Disease Classification System	

### 3.3 Safety

**Fig. 2:**
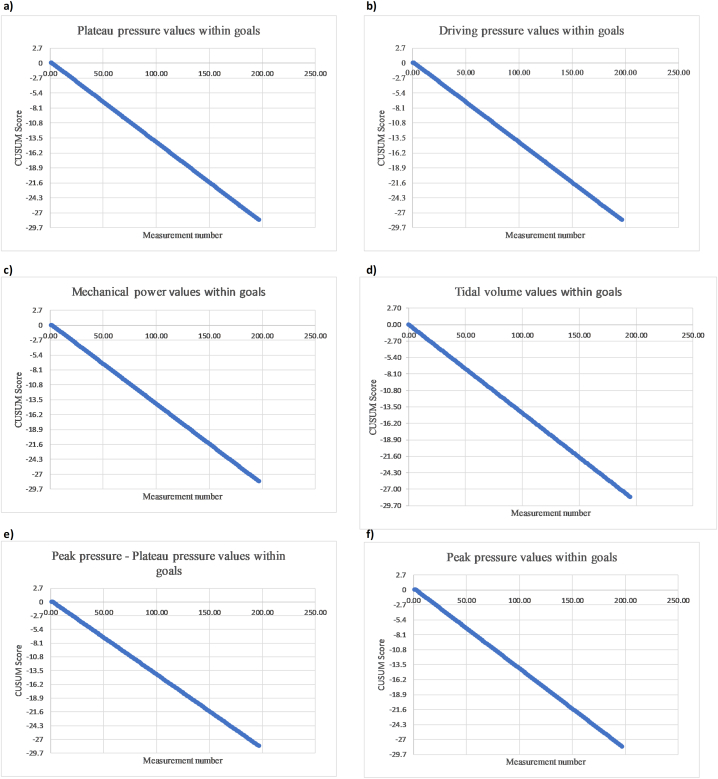
**CUSUM Safety Analysis.** The decision limits were set every 2.7; this value is calculated from the ranges of acceptable and unacceptable errors and the probabilities of type I and type II errors (see methods). A persistent trend of the curves crossing up to 10 decision limits from top to bottom can be seen, which indicates an adequate performance without events in which the parameters of protective ventilation were out of the safety ranges.

Safety assessment by CUSUM analysis of the protective ventilation parameters during the scheduled measurements showed curves that were persistently directed downward in all cases, crossing up to 10 decision limits in a top-down direction, without any events where the decision limit was crossed from the bottom-up or modifications in the direction or slope of the curve. This behavior indicates the absence of events in which the parameters related to lung protection protocols were out of the predefined safety ranges (Table S13 and Fig. 2A–F).

These safety parameters were maintained within the desired range of values in more than 98 % of continuous measurements, except during the procedures for aspiration of secretions or changes in position (including changes from supine to prone position), when the patients presented occasional episodes of variations in the monitored respiratory pressures (increase or decrease of these pressures related to the head mobilization, presence of the aspiration tube or the negative pressure required during suctioning), cough, desaturation, or decrease in blood pressure. These values represented less than 2 % of all continuous measurements; also, these were resolved within seconds after the corresponding intervention. Thus, they were classified as non-ventilator-related events.

Excluding the non-ventilator-related events, the peak pressure remained below 35 cmH2O, presenting the highest value at 32.6 cmH2O. The mechanical power was maintained below 15 J/min with a maximum value of 12.5 J/min. The plateau pressure was kept below 30 cm H2O, with the highest value of 22.5 cm H2O. Finally, the tidal volume was kept below 8 cc/Kg of ideal weight; its highest value was 7.5 cc/Kg (Table 2).

**Table 2:** Variability of ventilatory parameters in study patients.

Parameter	Min	Max	Median	P25	P75	Average	Standard deviation	Coefficient of variation
Tidal volume	315.0	533.0	438.0	416.0	461.0	428.2	55.2	0.1
Tidal volume per kg of ideal weight (cc/kg)	5.5	7.4	6.6	6.3	6.8	6.6	0.4	0.1
Minute ventilation (L)	4.8	10.4	7.9	7.3	8.5	7.6	1.3	0.2
Flow (L/min)	11.2	68.2	43.8	23.4	47.4	36.0	12.8	0.4
FiO2 (%)	30.0	80.0	35.0	30.0	40.0	37.0	8.4	0.2
Respiratory rate	14.9	20.8	17.6	17.5	18.5	17.7	1.1	0.1
PEEP (cmH2O)	6.5	11.2	8.0	7.8	8.3	8.1	0.6	0.1
I:E ratio insp	1.0	1.2	1.0	1.0	1.0	1.0	0.1	0.1
I:E ratio exp	1.0	2.4	2.1	2.1	2.2	1.9	0.5	0.3
Inspiratory time (sec.)	1.0	2.1	1.1	1.0	1.2	1.2	0.3	0.3
Expiratory time (sec.)	1.1	2.7	2.3	2.2	2.3	2.1	0.4	0.2
Static pulmonary compliance (ml/cmH2O)	22.6	74.9	38.7	35.1	42.3	37.9	8.6	0.2
Peak inspiratory pressure (maximum) (cmH2O)	20.0	32.6	24.8	23.0	26.7	25.1	2.9	0.1
Plateau Pressure	12.9	22.5	19.6	18.9	20.7	19.7	1.4	0.1
Driving pressure	5.5	14.5	11.3	10.9	12.6	11.6	1.5	0.1
Mechanical Power	3.7	12.6	8.6	7.5	9.2	8.5	1.3	0.1

Note: the I:E ratio was disaggregated into two lines to provide more information about this variable; the “I:E insp ratio” line shows the value that corresponds to inspiration, and the line “I: E exp ratio” shows the value that corresponds to expiration, so that a ratio I: E of 1:2 will be recorded with a value of “1″ in the variable “Ratio I: E insp” and a value of “2″ in the variable “Ratio I: E exp.” This table shows the central tendency and dispersion measures of ventilatory parameters measured every 15 min for the first 4 h and then every hour until 24 h.

**Fig. 3:**
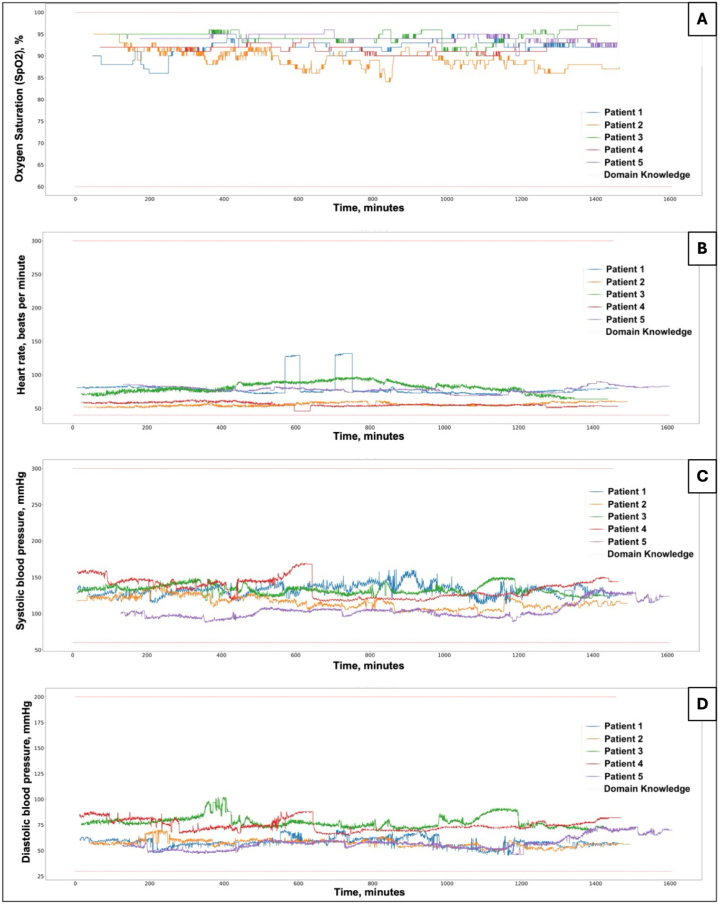
**Patient's vital signs during the study period.** Panel A shows the oxygen saturation; B, the heart rate; C, the systolic blood pressure; and D, the diastolic blood pressure.

During the experiments, the Unisabana-HERONS ventilators worked uninterrupted, with no failures and without the requirement for backup ventilators. Moreover, no clinical or radiological adverse events, such as ventilator-induced pneumothorax, pneumomediastinum, or subcutaneous emphysema, were detected in chest X-rays before or after their use. Notably, there were no ventilator-related episodes of hemodynamic deterioration. All vital signs were stable during the intervention period (Table 3 and Fig. 3A–D). Additionally, there were no episodes of cardiac arrest, death, elevated ureic nitrogen or creatinine, gastrointestinal bleeding, pneumonia, tracheobronchitis, or other ventilator-related adverse events in the observation period.

**Table 3:** Hemodynamic variables during continuous monitoring of vital signs.

VARIABLE		Patient 1	Patient 2	Patient 3	Patient 4	Patient 5
**Heart rate**	Min.	70.0	49.0	54.0	47.0	66.0
	1st qua.	74.0.	54.0	74.0	54.0	75.0
	Median	76.0	55.0	80.0	55.0	78.0
	Average	76.9	55.8	79.8	56.0	78.4
	3rd qua.	79.0	58.0	88.0	59.0	82.0
	Max.	91.0	66.0	108.0	66.0	92.0
**Systolic blood pressure**	Min.	111.0	89.0	115.0	88.0	86.0
	1st qua.	127.0	108.0	128.0	123.0	96.0
	Median	132.0	114.0	132.0	137.0	100.0
	Average	132.3	115.1	132.7	135.2	100.8
	3rd qua.	137.0	122.0	136.0	145.0	105.0
	Max.	154.0	141.0	150.0	179.0	121.0
**Diastolic blood pressure**	Min.	47.0	46.0	63.0	58.0	42.0
	1st qua.	56.0	54.0	73.0	70.0	52.0
	Median	58.0	57.0	76.0	73.0	56.0
	Average	58.0	56.9	76.8	74.9	55.7
	3rd qua.	61.0	59.0	79.0	79.0	58.0
	Max.	69.0	68.0	90.0	94.0	69.0

Notes: This table shows the measures of central tendency and dispersion of the hemodynamic variables obtained from the continuous recording of the vital signs monitored during the 24 h of use of the Herons ventilator. Min.: minimum; 1st qua.: first quartile; 3rd qua.: third quartile; Max.: maximum.

Nonetheless, one patient had a singular episode. This could be attributable to Vagus nerve irritation secondary to pharyngeal and glottic stimulation during orotracheal intubation [24]. Another patient presented a pattern of hydrostatic pulmonary edema in the chest x-ray, associated with a positive fluid balance of 1586 cc and urinary output of 0.38 cc/kg/hour. This event was managed with a reduction of water intake and the implementation of diuretics.

### 3.4 Effectiveness

Vital signs and arterial blood gases were analyzed to evaluate the effectiveness of the Unisabana-HERONS ventilator (Table 4). The median PaO2 (IQR) 30 min before connecting the patients to the ventilator was 80 mmHg (72.0–91.3), slightly above the ideal values. The median (IQR) oxygen saturation was 94 % (90.5–95.3), in the upper limit of the target values.

**Table 4:** Gas exchange and acid-base balance during the use of the Herons Ventilator.

Variable	Median 30 min pre-ventilator	IQR 30 min pre-ventilator	Range 30 min pre-ventilator	median 240 min	IQR 240 min	Range 240 min	Median 24 h	IQR 24 h.	Hr range. 24 hr	P min 30 pre ventilator vs min 240	P min 240 vs hr. 24	P min 30 pre-ventilator vs hr. 24
PaO2 mmHg	80.0	72.0–91.3	66.0–98.0	72.0	70.5–83.0	69.0–89.0	69.0	67.5–81.3	66.0–82.0	0.9	0.3	0.3
PaCO2 mmHg	40.0	38.8–40.5	38.0–42.0	40.0	37.3–42.5	35.0–44.0	38.0	34.8–42.0	34.0–54.0	1.0	0.6	0.9
SaO2 %	94.0	90.5–95.3	86.0–96.0	96.9	96.4–98.0	96.3–98.5	96.8	93.9–97.2	92.6–98.1	0.1	0.3	0.2
PaO2/FiO2	222.5	177.1–251.7	173.7–266.7	205.0	176.1–258.0	172.0–270.0	197.0	170.8–270.8	101.0–273.3	0.8	1.0	0.8
SaO2/FiO2	260.0	226.0–309.5	133.0–320.0	262.0	226.9–282.3	225.0–316.0	260.0	226.0–309.5	133.0–320.0	0.8	1.0	0.9
pH	7.39	7.38–7.44	7.36–7.45	7.4	7.39–7.41	7.38–7.42	7.46	7.37–7.48	7.34–7.49	0.8	0.3	0.4
BE	−0.8	(−) 2.5–2.8	(−) 2.6–3.9	−0.4	(−) 1.9–3.2	(−) 3.7–4.2	3.3	1.2–3.9	(−) 5.0–4.8	0.8	0.3	0.5
HCO3 mEq/L	24.0	22.8–26.5	22.0–28.0	24.9	22.9–27.5	20.9–28.5	26.8	24.6–28.6	19.8–29.3	0.8	0.8	0.3

Once the study was started, each patient's ventilator parameters were adjusted to reduce the PEEP and FiO2 levels to maintain the oxygenation range at the target values (Table S13), avoiding excessive supply of these parameters. Over time, the median PaO2 gradually decreases until it reaches the target range (55–70 mmHg). The difference between the median PaO2 at the beginning and the end of the intervention was 13.8 % due to the ventilator settings to avoid PaO2 greater than 70 mmHg. No decreases greater than 20 % or values below what was programmed were detected in the PaO2, with stability in PaO2 values and oxygen saturation during observation (Table 4).

Despite having included patients in whom a decrease in PaO2/FiO2 could have been expected due to disease progression, the median PaO2/FiO2 remained stable, with a decline of 11.5 % at the end of the 24 h of observation, this decrease was lower than the criterion of the clinical relevance of the study (reduction greater than 20 %) and was related with the underlying disease. The acid-base balance was stable, with similar PaCO2, HCO3, and pH levels, and within the clinical target ranges at the beginning and end of the intervention (Table 4).

### 3.5 Usability

The ventilator was rated as very easy to use, with a median response for all items between 9 and 10 with an IQR between 8 and 10 (Table S15).

## 4 Discussion

This was a preclinical and phase 1 clinical study of a low-cost ventilator, the Unisabana-HERONS, according to ISO standards. This device was tested on animals and humans with ARDS to analyze its safety, effectiveness, and usability. We demonstrated that the Unisabana-HERONS maintained an adequate gas exchange, allowing the delivery of lung protective ventilation using volume-controlled modes. The variability of the ventilatory parameters remained as programmed, with less than a 10 % difference among those measured by the Fluke VT900A gas flow analyzer gold standard. Finally, its ease of configuration and operation makes the Unisabana-HERONS ventilator an easy-to-use device, which is an advantage when training healthcare personnel non-expert personnel in LMIC, especially in the case of a state of emergency during the COVID-19 pandemic.

The lung protection protocol for volume-controlled ventilation has extensive support in the scientific literature due to its efficacy and safety [[25], [26], [27], [28], [29]]. Although manufactured low-cost pressure-control ventilators also had promising results [30], these strongly depend on respiratory compliance, have a variable tidal volume delivery, and increase volutrauma risk [31]. Thus, volume control was the primary mode in the Unisabana-HERONS. Our results in animals with ARDS showed that the ventilator proportionated an effective PEEP to increase the PaO2 levels and decrease the PaCO2 levels. Moreover, in the clinical phase, oxygen levels were stable and within the target limits; the plateau pressure, driving pressure, mechanical power, and tidal volume values provided by the ventilator ensured the lung-protecting ventilatory protocol, similar to the ARDS Network study [25], which also used volume-controlled ventilation. However, the PaO2/FiO2 of our patients was higher; this could be attributable to our exclusion of patients with PaO2/FiO2 <100. These outcomes assure the safety and efficacy of the Unisabana-HERONS ventilator.

The operation of the Unisabana-HERONS ventilator was configured to measure all the ventilatory parameters in real time. Sensors assessed the values displayed in the ventilator's screen with certified calibration to reduce the operation error and allow the identification of differences between the real and the settle parameters. A real-time visualization system is critical for data-driven decision-making during mechanical ventilation; it enhances the patient's treatment according to their needs (i.e., personalized medicine) and improves clinical outcomes in ARDS patients in the ICU [32,33]. The intra-subject, inter-subject, and ventilator parameters were kept within the aimed target range (Table S13) during the continuous monitoring. Also, the CUSUM analysis demonstrated [[17], [18], [19]] no deviations from the protocol goals for ventilatory parameters. A comparable technology was used in the SERKAN ventilator, which was also developed during the COVID-19 pandemic and was categorized by physicians as complete, intuitive, practical, and straightforward [34]. In our study, healthcare workers also classified the Unisabana-HERONS ventilator as easy to access and an intuitive device that does not require the operator to navigate the complex menus of the computerized operating systems.

All the discussed characteristics of the Unisabana-HERONS confirm that it is similar to high-tech commercial ventilators. Unlike other medical devices, ventilators cannot be produced in mass, and only a few companies have the expertise and permission to build them. Thus, accessing these expensive health technologies is a significant challenge for LMICs, and for many years, they have been working with under-resourced, overstretched, and overwhelmed health systems [9,13,14]. Therefore, developing rapidly manufactured, cost-effective technologies with robust evidence should be prioritized for these countries [[35], [36], [37], [38]]. Moreover, it can bring essential medical goods to LMIC and eliminate barriers and disparities in access to good quality healthcare [39,40]. Low-cost medical devices such as the Unisabana-HERONS ventilator demonstrated that it is possible to create high-quality devices that reach the ISO standard, proportionate adequate treatment to patients, and could be easily and rapidly acquired by LMICs. It is essential to highlight that even the Unisabana-HERONS is not commercially available, and thus, we could not perform a complete cost-effectiveness analysis; the production cost of this ventilator is less than 3000 USD, which is ten times lower than the average cost of a high-tech ventilator in Colombia.

There are some limitations and strengths in this study that should be recognized. It was possible to observe the non-comparative behavior of the Unisabana-HERONS ventilator with favorable results in terms of effectiveness, safety, and ease of use in providing volume-controlled mechanical ventilation. However, its non-comparative design does not allow definitive inferences about its clinical equivalence with other legally approved ventilators. This could only be done through a comparative study of substantial equivalence, ideally under a crossover design. Nevertheless, the Unisabana-HERONS ventilator was a helpful tool to support patients with varied respiratory failure causes in situations with limited resources. Even more, it is a low-cost medical device. Moreover, it is crucial to recognize that the Unisabana-HERONS was only used in a few subjects, and the limited sample size of this study is an important limitation. More studies of cost-effectiveness analysis are required to demonstrate its advantages in cost outcomes compared with commercial ventilators.

The Unisabana-Herons was proved to be a low-cost ventilator that met the ISO standards. It effectively provided adequate gas exchange, maintained the ventilatory parameters in the range of lung protection ventilatory strategies, and displayed the settled physiological parameters in real-time; thus, the personnel determined it to be easy to use. These characteristics make the Unisabana-HERONS ventilator a safe and efficient device for ARF patients that could also benefit the reduction of technological access barriers to healthcare in LMICs such as Colombia.

## Funding

This study was funded by 10.13039/501100010628Universidad de La Sabana under the grant MED-328-2022.

## Data availability

All the data generated in the study are available after a reasonable request to the corresponding author.

## CRediT authorship contribution statement

**Julian Echeverry:** Writing – review & editing, Writing – original draft, Visualization, Validation, Methodology, Luis Alfredo Paipa, Writing – review & editing, Writing – original draft, Visualization, Validation, Methodology, Juan Carlos Camelo, Writing – review & editing, Writing – original draft, Visualization, Validation, Methodology, Laura Cucunubo, Writing – review & editing, Writing – original draft, Visualization, Validation, Methodology, Santiago Pedraza, Writing – review & editing, Writing – original draft, Visualization, Validation, Methodology, Investigation, Fabio Varón-Vega, Writing – review & editing, Writing – original draft, Visualization, Validation, Supervision, Software, Resources, Project administration, Methodology, Investigation, Data curation, Conceptualization, Luis Reyes, Writing – review & editing, Writing – original draft, Visualization, Validation, Supervision, Software, Resources, Project administration, Methodology, Investigation, Formal analysis, Data curation, Conceptualization, Alirio Bastidas, Writing – review & editing, Writing – original draft, Visualization, Validation, Supervision, Software, Methodology, Investigation, Data curation, Conceptualization, Ronaldo Roncancio Rachid, Writing – review & editing, Writing – original draft, Visualization, Validation, Project administration, Methodology, Luis Fernando Giraldo-Cadavid, Writing – review & editing, Writing – original draft, Visualization, Validation, Supervision, Software, Resources, Project administration, Methodology, Investigation, Funding acquisition, Formal analysis, Data curation, Conceptualization, Rubén Darío Henao I, Writing – review & editing, Writing – original draft, Visualization, Validation, Methodology. **Fabio Varón-Vega:** Writing – review & editing, Writing – original draft, Visualization, Validation, Methodology, Elsa D. Ibáñez-Prada, Writing – review & editing, Writing – original draft, Visualization, Validation, Methodology, Iván Arturo Ramírez, Writing – review & editing, Writing – original draft, Visualization, Validation, Methodology, Investigation, Esteban García-Gallo, Writing – review & editing, Writing – original draft, Visualization, Validation, Software, Methodology, Formal analysis, Diego Nicolás Rincón, Writing – review & editing, Writing – original draft, Visualization, Validation, Methodology, Investigation, Henry Oliveros, Writing – review & editing, Writing – original draft, Visualization, Validation, Methodology, Investigation, Formal analysis.

## Declaration of competing interest

The authors declare the following financial interests/personal relationships which may be considered as potential competing interests: Luis Fernando Giraldo-Cadavid reports administrative support, equipment, drugs, or supplies, statistical analysis, and writing assistance were provided by 10.13039/501100010628Universidad de La Sabana.
