# Supplementary material for: The development and implementation of a low-cost mechanical ventilator in a low-middle-income country during the COVID-19 pandemic: The Unisabana-HERONS
Source: Heliyon. 2024 May 5;10(9):e30671. doi: 10.1016/j.heliyon.2024.e30671 (PMC11096896; doi:10.1016/j.heliyon.2024.e30671)
Supplement: Multimedia component 1 [file mmc1.docx]

# **ONLINE SUPPLEMENT**

## **1. ADDITIONAL INFORMATION**

## **1.1 PROTOTYPE TESTING REPORT OF THE UNISABANA VENTILATOR PROTOTYPE - HERONS**

### 1.1.1 Activity 1

Ventilator testing of Unisabana- HERONS mechanical ventilator prototype with ASL 5000 respiratory simulator and Ingmar medical.

Date: June 15 to 16, 2020

Time: 6:00 a.m. on June 15/- 18:00 hours on June 16

Place: Center for training and research in mechanical ventilation CEIVEM - Colombian Pneumological Foundation.

The Unisabana-HERONS ventilator is evaluated after the tests performed on the ASL 5000 simulator and PB-980 conventional ventilator, it was settled the same parameters as the previous test were developed.

1. The ventilator is connected to the medical gases point (air and oxygen), and proper operation and coupling to the system were checked.

2. Ventilatory programming is started, settling parameters for protective mechanical ventilation (tidal volume (VC) of 6 to 8 cc/kg of ideal weight), respiratory frequency (FR): 14 rpm, fraction of inspired oxygen (FiO2): 21%, positive end-expiratory pressure (PEEP): 8 cmH2O, inspiration: expiration ratio (I:E) 1:2.3. The cycle was tested for 2 hours connected to the simulator which is programmed in this initial phase with values of normal pulmonary ventilation.

3. The Puritan Bennett 980 ventilator was connected, to the IngMar medical test lung with established parameters, calculating an ideal weight of 66 kg. Initially, Resistance (R) was programmed at 5 cm/H2O/l/sec, Distensibility (D) or Compliance of 40ml/cmH20, and the following variables are recorded: Peak Pressure (PP), Plateau Pressure (PM) and Total Exhaled Volume (TEV). Subsequently, the resistance was raised to 20 cm/H2O/l/sec and 50 cm/H2O/l/sec, with distensibility at 20 and 40ml/cmH20, obtaining the same recording of the established variables. These measurements were taken on 5 occasions.

Subsequently, the ventilator of the University of La Sabana-HERONS was connected, performing the same tests with the IngMar medical test lung (resistance and distensibility variations), and these measurements were carried out on 5 occasions. The results are presented in Tables 1 to 5 with central tendency measures.

**Table S1.**

**Results with the use of the Ingmar medical simulator, Tidal volume of 400 ml, R at 5 cm/H2O/l/sec; D 40ml/cmH20 (5 measures)**

| **Variable** | | **PB 980 ventilator** | **Unisabana-HERONS ventilator** |
| --- | --- | --- | --- |
| **PP cmH_2_O** | |  | |
|  | **Median** | 19.0 | 19.0 |
|  | **Mean** | 18.6 | 18.6 |
|  | **Desviation** | 0.4277 | 0.5477 |
| **PM mH_2_O** | |  | |
|  | **Median** | 12.0 | 12.0 |
|  | **Mean** | 12.0 | 12.2 |
|  | **Desviation** | 0 | 0 |
| **VTE ml** | |  | |
|  | **Median** | 401.0 | 400.0 |
|  | **Mean** | 401.4 | 399.8 |
|  | **Desviation** | 1.1401 | 1.7888 |

**Table S2.**

**Results with the use of the Ingmar medical simulator, Tidal volume of 400 ml, R at 20 cm/H2O/l/sec; D 20ml/cmH20 (5 measures)**

| **Variable** | | **PB 980 ventilator** | **Unisabana-HERONS ventilator** |
| --- | --- | --- | --- |
| **PP cmH_2_O** | |  | |
|  | **Median** | 22.0 | 22.0 |
|  | **Mean** | 22.0 | 22.4 |
|  | **Desviation** | 0 | 0.5477 |
| **PM mH_2_O** | |  | |
|  | **Median** | 14.0 | 14.0 |
|  | **Mean** | 13.6 | 13.8 |
|  | **Desviation** | 0.5477 | 0.4472 |
| **VTE ml** | |  | |
|  | **Median** | 400.0 | 399.0 |
|  | **Mean** | 400.0 | 398.8 |
|  | **Desviation** | 0 | 1.3038 |

**Table S3.**

**Results with the use of the Ingmar medical simulator, Tidal volume of 400 ml, R at 50 cm/H2O/l/sec; D 20ml/cmH20 (5 measures)**

| **Variable** | | **PB 980 ventilator** | **Unisabana-HERONS ventilator** |
| --- | --- | --- | --- |
| **PP cmH_2_O** | |  | |
|  | **Median** | 27.0 | 27.0 |
|  | **Mean** | 26.8 | 27.0 |
|  | **Desviation** | 0.4472 | 0.7071 |
| **PM mH_2_O** | |  | |
|  | **Median** | 19.0 | 20.0 |
|  | **Mean** | 19.6 | 19.8 |
|  | **Desviation** | 0.8944 | 0.4472 |
| **VTE ml** | |  | |
|  | **Median** | 396.0 | 392.0 |
|  | **Mean** | 395.8 | 392.4 |
|  | **Desviation** | 1.7888 | 2,6076 |

**Table S4.**

**Results with the use of the Ingmar medical simulator, Tidal volume of 400 ml, R at 20 cm/H2O/l/sec; D 40ml/cmH20 (5 measures)**

| **Variable** | | **PB 980 ventilator** | **Unisabana-HERONS ventilator** |
| --- | --- | --- | --- |
| **PP cmH_2_O** | |  | |
|  | **Median** | 22.0 | 22.0 |
|  | **Mean** | 22.0 | 21.8 |
|  | **Desviation** | 0 | 0.14472 |
| **PM mH_2_O** | |  | |
|  | **Median** | 14.0 | 13.0 |
|  | **Mean** | 14.0 | 13.2 |
|  | **Desviation** | 0 | 0.4472 |
| **VTE ml** | |  | |
|  | **Median** | 400.0 | 400.0 |
|  | **Mean** | 400.0 | 399.4 |
|  | **Desviation** | 0 | 0.8944 |

**Table S5.**

**Results with the use of the Ingmar medical simulator, Tidal volume of 400 ml, R at 50 cm/H2O/l/sec; D 20ml/cmH20 (5 measures)**

| **Variable** | | **PB 980 ventilator** | **Unisabana-HERONS ventilator** |
| --- | --- | --- | --- |
| **PP cmH_2_O** | |  | |
|  | **Median** | 28.0 | 29.0 |
|  | **Mean** | 28.2 | 29.4 |
|  | **Desviation** | 0.4472 | 0.5477 |
| **PM mH_2_O** | |  | |
|  | **Median** | 22.0 | 22.0 |
|  | **Mean** | 21.8 | 21.8 |
|  | **Desviation** | 0.4472 | 0.4472 |
| **VTE ml** | |  | |
|  | **Median** | 400.0 | 398.0 |
|  | **Mean** | 400.0 | 387.8 |
|  | **Desviation** | 0 | 1.0954 |

After performing this test, the prototype of the Unisabana-HERONS mechanical ventilator is connected to the Respiratory Simulation Specialists IngMar medical ASL 5000, through an artificial airway with TOT number 8.0. It was tested with an initial program for normal respiratory ventilation and later evaluated with variations in PP, PM, and VTE.

Comparisons were made between the variations found in the PB 980 ventilator and the Unisabana-HERONS ventilator. The results are presented in Tables 6 to 10 with central tendency measures.

Programming:

Ideal weight: 66 kg

Tidal volume: 400 ml

R settle: 5 cm/H2O/l/sec

D settle: 50 ml/cmH20

**Table S6.**

**Results using the Respiratory Simulation Specialists IngMar medical ASL 5000, Tidal volume of 400 ml, R at 5 cm/H2O/l/sec; D 50 ml/cmH20**

| **Variable** | | **PB 980 ventilator** | **Unisabana-HERONS ventilator** |
| --- | --- | --- | --- |
| **PP cmH_2_O** | |  | |
|  | **Median** | 14.0 | 14.0 |
|  | **Mean** | 14.0 | 14.0 |
|  | **Desviation** | 0 | 0.4472 |
| **PM mH_2_O** | |  | |
|  | **Median** | 8.0 | 8.0 |
|  | **Mean** | 8.0 | 8.4 |
|  | **Desviation** | 0 | 0.5477 |
| **VTE ml** | |  | |
|  | **Median** | 400.0 | 399.0 |
|  | **Mean** | 400.0 | 399.4 |
|  | **Desviation** | 0 | 1.6733 |

**Table S7.**

**Results using the Respiratory Simulation Specialists IngMar medical ASL 5000, Tidal volume of 400 ml, R at 100 cm/H2O/l/sec; D 10 ml/cmH20**

| **Variable** | | **PB 980 ventilator** | **Unisabana-HERONS ventilator** |
| --- | --- | --- | --- |
| **PP cmH_2_O** | |  | |
|  | **Median** | 61.0 | 62.0 |
|  | **Mean** | 61.0 | 62.4 |
|  | **Desviation** | 1 | 0.8944 |
| **PM mH_2_O** | |  | |
|  | **Median** | 36.0 | 34.0 |
|  | **Mean** | 35.6 | 34.2 |
|  | **Desviation** | 0.8944 | 0.4472 |
| **VTE ml** | |  | |
|  | **Median** | 374.0 | 382.0 |
|  | **Mean** | 374.8 | 381.2 |
|  | **Desviation** | 3.3466 | 3.0331 |

**Table S8.**

**Results using the Respiratory Simulation Specialists IngMar medical ASL 5000, Tidal volume of 400 ml, R at 50 cm/H2O/l/sec; D 10 ml/cmH20**

| **Variable** | | **PB 980 ventilator** | **Unisabana-HERONS ventilator** |
| --- | --- | --- | --- |
| **PP cmH_2_O** | |  | |
|  | **Median** | 46.0 | 46.0 |
|  | **Mean** | 45.6 | 46.2 |
|  | **Desviation** | 0.5477 | 1.0954 |
| **PM mH_2_O** | |  | |
|  | **Median** | 32.0 | 32.0 |
|  | **Mean** | 31.2 | 32.0 |
|  | **Desviation** | 1.0954 | 1.4142 |
| **VTE ml** | |  | |
|  | **Median** | 384.0 | 388.0 |
|  | **Mean** | 383.2 | 387.2 |
|  | **Desviation** | 1.788 | 1.0954 |

**Table S9.**

**Results using the Respiratory Simulation Specialists IngMar medical ASL 5000, Tidal volume of 400 ml, R at 25 cm/H2O/l/sec; D 50 ml/cmH20**

| **Variable** | | **PB 980 ventilator** | **Unisabana-HERONS ventilator** |
| --- | --- | --- | --- |
| **PP cmH_2_O** | |  | |
|  | **Median** | 40.0 | 40.0 |
|  | **Mean** | 39.4 | 40.8 |
|  | **Desviation** | 0.8944 | 1.0954 |
| **PM mH_2_O** | |  | |
|  | **Median** | 22.0 | 22.0 |
|  | **Mean** | 22.0 | 21.6 |
|  | **Desviation** | 0 | 0.5477 |
| **VTE ml** | |  | |
|  | **Median** | 400.0 | 396.0 |
|  | **Mean** | 400.4 | 395.2 |
|  | **Desviation** | 0.8944 | 3.0331 |

Finally, a simulation is performed on an adult patient with acute respiratory distress syndrome (ARDS) with mild, moderate, and severe severity. It was programmed dynamic ventilatory parameters in the prototype. A correlation between pressures and volumes was obtained by the monitoring of the prototype and the simulator (movement visualization equation). It should be noted that for this simulation the variation performed in the simulator is evaluated and compared with the change presented in the ventilator. The Unisabana-HERONS ventilator was programmed with tidal volume (VC) and positive end-expiratory pressure (PEEP) then measurements were performed in the simulator to evaluate their correlation, there wasn´t any failure in the structure of function during the test performed, so the test was finished without inconvenient. The results are presented in Table 10-12 with central tendency measures.

**Table S10.**

**Results using the Respiratory Simulation Specialists IngMar medical ASL 5000, mild ARDS clinical scenario.**

| **Variable** | | **Unisabana-HERONS ventilator** | **Simulator ASL 5000** |
| --- | --- | --- | --- |
| **VC (ml)** | |  | |
|  | **Median** | 450 | 461.0 |
|  | **Mean** | 450 | 460.8 |
|  | **Deviation** | 129.0994 | 136.5512 |
| **PEEP (cmHO2)** | |  | |
|  | **Median** | 17.5 | 17.5 |
|  | **Mean** | 17.5 | 17.5 |
|  | **Deviation** | 6,4549 | 5.6862 |

**Table S11.**

**Results using the Respiratory Simulation Specialists IngMar medical ASL 5000, moderate ARDS clinical scenario.**

| **Variable** | | **Unisabana-HERONS ventilator** | **Simulator ASL 5000** |
| --- | --- | --- | --- |
| **VC (ml)** | |  | |
|  | **Median** | 450 | 453.0 |
|  | **Mean** | 450 | 454.5 |
|  | **Deviation** | 129.0994 | 130.9185 |
| **PEEP (cmHO2)** | |  | |
|  | **Median** | 17.5 | 16.5 |
|  | **Mean** | 17.5 | 17.75 |
|  | **Deviation** | 6,4549 | 5.8523 |

**Table S12.**

**Results using the Respiratory Simulation Specialists IngMar medical ASL 5000, severe ARDS clinical scenario.**

| **Variable** | | **Unisabana-HERONS ventilator** | **Simulator ASL 5000** |
| --- | --- | --- | --- |
| **VC (ml)** | |  | |
|  | **Median** | 450 | 442.0 |
|  | **Mean** | 450 | 442.5 |
|  | **DDeviation** | 129.0994 | 130.4977 |
| **PEEP (cmHO2)** | |  | |
|  | **Median** | 17.5 | 17.0 |
|  | **Mean** | 17.5 | 17.0 |
|  | **DDeviation** | 6,4549 | 5.9441 |

### 1.1.2 Activity 2

Functional test

Date: June 15-16, 2020

Time: 6:00 a.m. on June 15/- 6:00 p.m. on June 16

Place: Center for training and research in mechanical ventilation CEIVEM - Colombian Pneumological Foundation.

1. Mechanical ventilator is connected to medical gas points, and IngMar medical test lung with resistance of 20 and distensibility of 20.

2. The following ventilatory parameters were settled: flow 32 ml and it was obtained a tidal volume: 380/400 ml, PEEP: 8.0 cmH2O, respiratory frequency: 14 rpm, inspiratory time: 0.9 s, I:E ratio 1:2.3, FiO2: 21 %.

3. An adequate cycling and dynamic ventilatory monitoring were observed and the pressure/time and flow/time curves were recorded.

4. Records were taken at different times on the mentioned days. The Unisabana-HERONS ventilator functioned properly without overheating and adequate ventilatory monitoring according to the ventilatory parameters adjustments made during each visit.

The test finished without any inconvenience.

The effectiveness outcomes to evaluate were:

1. Improvement or maintenance of the oxygenation level measured by PaO2 and O2 Saturation (SaO2). Maintenance was defined as a decrease of less than 20% in the levels of PaO2 and SaO2 with respect to the baseline measurements made in the 30 minutes before connecting the Unisabana-HERONS

ventilator.

2. Improvement or maintenance of adequate levels of carbon dioxide measured by PaCO2. Maintenance was defined as a change of less than 20% in the levels of PaCO2 with respect to the baseline measurements made in the 30 minutes before connecting the Unisabana-HERONS ventilator.

3. Improvement or stability of PaO2 / FiO2 and SaO2 / FiO2 during ventilation. Stability was defined as a decrease of less than 20% in these variables with respect to the baseline measurements made in the 30 minutes before connecting the Unisabana-HERONS ventilator.

The safety outcomes to be evaluated were:

1. Uninterrupted operation and without failures in the period of use of the ventilator.

2. Peak inspiratory pressure (PIP) > 35 cm H2O that does not have a clinical explanation other than the ventilator. Examples of causes of peak inspiratory pressure elevation other than the ventilator included mucus plugs, kinking of the orotracheal tube during a change in position, or the presence of a secretion aspiration tube or other type of foreign body in the orotracheal tube.

3. Plateau airway pressure > 30 cm H2O that has no clinical explanation other than the ventilator. Examples of causes of plateau pressure elevation other than the ventilator included progression of ARDS, orotracheal tube kinking during a change in position, or the presence of an aspiration tube or any other type of foreign body in the orotracheal tube.

4. TV > 8 cc/kg of ideal weight that does not have a clinical explanation other than the ventilator (such as severe hypercapnia due to bronchospasm)

5. Unscheduled decrease or increase in respiratory rate, tidal volume, PEEP, peak inspiratory pressure, FiO2.

6. Pneumothorax, pneumomediastinum, subcutaneous emphysema (that do not have an explanation other than ventilatory support such as the insertion of a central catheter or airway wounds)

Usability was evaluated using a standardized questionnaire where people who were operating the ventilator after receiving theoretical training (eg. respiratory therapist, doctor) were asked to rate the following items on a numerical scale going from 0 to 10 according to the following guide:

1. Easy to set the ventilator tidal volume:

0: it was impossible to set the tidal volume; 10: it was extremely easy to set the tidal volume

2. Easy to set the ventilator's respiratory rate:

0: It was impossible to set the respiratory rate; 10: It was extremely easy to set the respiratory rate

3. Easy to set the inspired fraction of oxygen from the ventilator:

0: It was impossible to set the inspired fraction of oxygen; 10: It was extremely easy to set the inspired fraction of oxygen

4. Easy to set the PEEP (positive pressure at the end of expiration) of the ventilator:

0: it was impossible to set the PEEP (positive pressure at the end of expiration); 10: it was extremely easy to set the PEEP (positive pressure at the end of expiration)

5. Easy to set the inspiratory time of the ventilator:

0: It was impossible to set the inspiratory time; 10: It was extremely easy to set the inspiratory time.

6. Ease of setting the ventilator inspiration: expiration ratio:

0: It was impossible to configure the inspiration: expiration ratio; 10: It was extremely easy to set up the inspiration: expiration ratio

7. Easy to configure ventilator alarms:

0: it was impossible to configure the alarms; 10: it was extremely easy to set the alarms

8. Easy to connect the circuits that carry the air from the ventilator to the patient and the necessary filters:

0: It was impossible to connect the circuits that carry the air from the ventilator to the patient and the necessary filters; 10: It was extremely easy to set up connecting the circuits that carry air from the ventilator to the patient and the necessary filters

9. Ease of connecting the ventilator to oxygen and air sources (hospital gas pipes, gas cylinders, compressor):

0: It was impossible to connect the ventilator to the oxygen and air sources; 10: It was extremely easy to connect the ventilator to the oxygen and air sources

## **1.2 CUSUM ANALYSIS**

A binary cumulative sum analysis (CUSUM analysis) was performed to assess the frequency with which ventilatory parameters were found to be outside the safety ranges detailed in Table 1 (out of control). This method allows us to continuously monitor a production process, the competence of a professional, or the performance of a device to detect subtle deviations from a previously defined level of good performance. The CUSUM analysis is characterized by having high sensitivity and specificity to detect events with punctual and subtle deviations from the pre-established quality criteria [1, 2].

When a ventilatory parameter was outside the safety ranges indicated in Table 1, it was considered a failure, and when it was within the safety ranges, it was considered a success. In this way, each measurement of the security parameters is binary classified as success (within the safety range) or failure (outside the safety range). In the CUSUM analyses, all the specific measurements carried out for each variable at the moments established in the study protocol were included. Such moments were every 15 minutes from 30 minutes before the start of ventilation with the Unisabana-Herons ventilator until hour 4 (minute 240) of use of the Herons ventilator and every hour in the next 20 hours.

We considered an acceptable error rate when 10% of the measurements were outside the safety ranges (**Table 1**) and unacceptable when 20% or more of the measurements were outside those ranges. Such error rates are commonly used in procedures where 90% or more of events qualified as success are considered optimal [1, 2]. We defined a Type I error (probability of falsely classifying the ventilator as poor performance, designated α) of 0.1 and a Type II error (probability of falsely classifying the ventilator as good performance, designated β) of 0.1 according to the usual recommendations for this method [1, 2].

We plotted the CUSUM binomial graphs using Microsoft Excel 2013 (Microsoft Corporation, Redmond, WA, USA). This method consists of the cumulative sum of failures minus successes in each case. We draw the CUSUM curve by plotting the cumulative sum score after each case (y-axis) versus that case's index number (x-axis). Consecutive failures drive the CUSUM curve up, while consecutive successes drive the CUSUM curve down. The CUSUM graph includes horizontal lines called decision limits (h1 and h0), which are the limits of an acceptable or unacceptable error rate and are calculated based on the risk of α and β errors. When the CUSUM curve crosses a decision limit from up to down, it is inferred that the failure rate was within the acceptable performance range of less than 10% failures (good performance); when the CUSUM curve crosses a decision limit from down to up, it is inferred that the failure rate has reached the unacceptable value of 20% (bad performance); if the CUSUM curve remains between two decision limits, continuous observation (stable performance) is indicated. Therefore, good performance is assumed when the CUSUM curve slopes downward or remains stable, but when the curve slopes upward, it indicates a less-than-acceptable success rate.

## **2. SUPPLEMENTARY TABLES**

**Table S13**. Gas Exchange and ventilatory parameters goals for each patient.

| **Variable** | **Units** | **Lower limit** | **Higher Limit** |
| --- | --- | --- | --- |
| PaO2 | mmHg | 55 | 70 |
| PaCO2 | mmHg | 32 | 60 |
| pH |  | 7.2 | 7.5 |
| SaO2 | % | 84 | 94 |
| Respiratory rate | bpm | 12 | 30 |
| FiO2 |  | 0.3 | 1 |
| **Ventilatory parameters** | | | |
| Tidal volume (TV) | mL/kg | 4 | 8 |
| Plateau pressure | cm H2O | N.A. | 30 |
| Driving pressure (plateau pressure - PEEP) | cm H2O | N.A. | 15 |
| PEEP | cm H2O | 5 | 22 |
| PIP | cm H2O | 10 | 70 |
| Mechanical power | J/min | N.A. | 15 |
| *Consider weaning of ventilatory support if: SaO2>90% with FiO2 < 0.4 and PEEP<8cmH2O | | | |

Abbreviations: N.A.: not applicable; bpm: breaths per minute; PEEP: positive end-expiratory pressure; PIP: peak inspiratory pressure. These goals were adopted for Bogota (situated 2,600 meters above sea level) [3]

**Table S14**. Interventions and measures in the animal model used to evaluate Unisabana-HERONS ventilator.

| Time | Estate (healthy vs ARDS) | Ventilator | Intervention | VT (mL) | PEEP (cm H2O) | Volumen min (L/min) | Plateau pressure  (cmH2O) | Airway median pressure (cmH2O) | PaO2 (mmHg) | PaCO2 (mmHg) | PaFi | pH | PIP (cmH2O) | FiO2 | RR | AP (mmHg) | HR (bpm) | SatO2 | Insp.  t (seg) | Exp  t (seg) | Cycle  t (seg) | Insp  Flow (L/min) | Distensibility (mL/cmH2O) |
| --- | --- | --- | --- | --- | --- | --- | --- | --- | --- | --- | --- | --- | --- | --- | --- | --- | --- | --- | --- | --- | --- | --- | --- |
| 11:30 | HEALTHY | Surgivet cds 9000 | General anesthesia | 300 | 5 |  | 20 |  |  |  |  |  |  |  | 19 | 122/81 | 96 | 98 | 1 | 1 | 2 |  | 20.0 |
| 11:46 | HEALTHY | Herons | General anesthesia | 300 | 7 | 5.9 | 16.6 | 9.2 | 171.2 | 57.1 (40) | 171 | 7.35 | 20.8 | 100 | 20 | 101/78 | 86 | 99.4 | 1 | 2.1 | 3.1 | 39 | 31.3 |
| 12:00 | HEALTHY | Herons | General anesthesia | 380 | 12 | 7.4 | 25.9 | 15.0 |  |  |  |  | 30.5 | 100 | 19 | 122/95 | 98 | 99.5 | 1 | 2.1 | 3.1 | 47.1 | 27.3 |
| 12:11 | HEALTHY | Herons | General anesthesia | 443 | 15 | 7.8 | 23.4 | 17.7 | 467.3 | 40.5 | 467 | 7.45 | 32 | 100 | 17 | 106/80 | 90 | 99.4 | 1.1 | 2.4 | 3.5 | 54.4 | 52.7 |
|  |  |  |  |  |  |  |  |  |  |  |  |  |  |  |  |  |  |  |  |  |  |  |  |
| 12:48 | SDRA  Induction | Herons | NSS  Lavage  30 cc/kg | 700 | 5 | 9.1 | 30.5 | 10.2 | 110.1 | 29.2 | 314.2 | 7.51 | 37.8 | 35 | 13 | 103/77 | 84 | 96 | 1.5 | 3.2 | 4.7 | 45.7 | 27.5 |
| 13:45 | ALI | Herons | NSS  Lavage  30 cc/kg | 734 | 4 | 9.5 | 34 | 10.4 | 91.5 | 28.9 | 183 | 7.47 | 43.8 | 50 | 13 | 119/95 | 98 | 95 | 1.5 | 3.2 | 4.7 | 52.2 | 24.5 |
| 14:35 | ARDS | Herons | NSS  Lavage  30 cc/kg | 866 | 4 | 10.7 | 40 | 11.4 | 84.3 | 32.6 | 84.3 | 7.4 | 50.4 | 100 | 13 | 100/77 | 120 | 94 | 1.5 | 3.2 | 4.7 | 57.1 | 24.1 |
|  |  |  |  |  |  |  |  |  |  |  |  |  |  |  |  |  |  |  |  |  |  |  |  |
| 14:45 | ARDS | Herons | Observation | 380 | 10 | 6.1 | 32 | 14.0 |  |  |  |  | 34.7 | 100 | 16 | 135/107 | 108 | 82 | 1.2 | 2.5 | 3.7 | 34.5 | 17.3 |
| 15:02 | ARDS | Herons | Ventilator adjustment | 380 | 10 | 6.1 | 32 | 14.0 | 53.2 | 67.6 | 53.2 | 7.1 | 34.7 | 100 | 16 | 105/90 | 123 | 90 | 1.2 | 2.5 | 3.7 | 34.5 | 17.3 |
| 15:15 | ARDS | Herons | Ventilator adjustment | 390 | 14 | 6.9 | 34 | 19.5 |  |  |  |  | 36 | 100 | 17 | 118/91 | 135 | 90 | 1.7 | 1.7 | 3.4 | 19.2 | 19.5 |
| 15:31 | ARDS | Herons | Ventilator adjustment | 421 | 13 | 9.3 | 35.4 | 20.5 | 71.6 | 55.7 | 71.6 | 7.2 | 36.8 | 100 | 22 | 115/85 | 149 | 88 | 1.7 | 1 | 2.7 | 21.2 | 18.8 |
| 15:50 | ARDS | Herons | Ventilator adjustment | 337 | 20 | 6.5 | 37.4 | 23.8 | 124 | 61.8 | 165.3 | 7.13 | 39.1 | 75 | 20 | 94/67 | 170 | 97 | 1.2 | 1.8 | 3 | 30.4 | 19.4 |
| 16:30 |  |  | EUTHANASIA |  |  |  |  |  |  |  |  |  |  |  |  |  |  |  |  |  |  |  |  |

Notes: PaFi: PaO2/FiO2, ARDS: acute respiratory distress syndrome, TV: tidal volume, PEEP: positive end-expiratory pressure, PIP: peak inspiratory pressure, FiO2: fraction of inspired oxygen, RR: respiratory rate per minute, AT: arterial tension; HR: heart rate per minute, SatO2: oxygen saturation by pulse oximetry, Insp t: inspiratory time; Exp t: expiratory time; Cycle t: total time of respiratory cycle, Insp flow: inspiratory Flow

**Table S15**. Usability Questionnaire.

| **Question** | **N** | **Median** | **IQR** |
| --- | --- | --- | --- |
| Easy to set the tidal volume | 16 | 9 | 8 .0-10.0 |
| Ease of setting the respiratory rate | 16 | 10 | 9.3-10.0 |
| Easy to configure the inspired oxygen fraction | 16 | 10 | 10.0-10.0 |
| Easy to configure the PEEP (positive pressure at the end of expiration) | 16 | 10 | 9.0-10.0 |
| Easy to set inspiratory time | 16 | 10 | 9.0-10.0 |
| Easy to configure the inspiratory: expiratory ratio (I: E) | 16 | 10 | 9.0-10.0 |
| Easy to set alarms | 16 | 10 | 9.0-10.0 |
| Easy to connect the circuits that carry the gas from the ventilator to the patient and the filters | 16 | 10 | 10.0-10.0 |
| Easy to connect the ventilator to gas sources (pipes, cylinders...) | 16 | 9 | 9.0-9.0 |
| Notes: N: number of people who answered the questionnaire, IQR: interquartile range (25th percentile to 75th percentile) | | | |

## **3. SUPPLEMENTARY FIGURES**

**Figure S1**. CONSORT Flowchart.


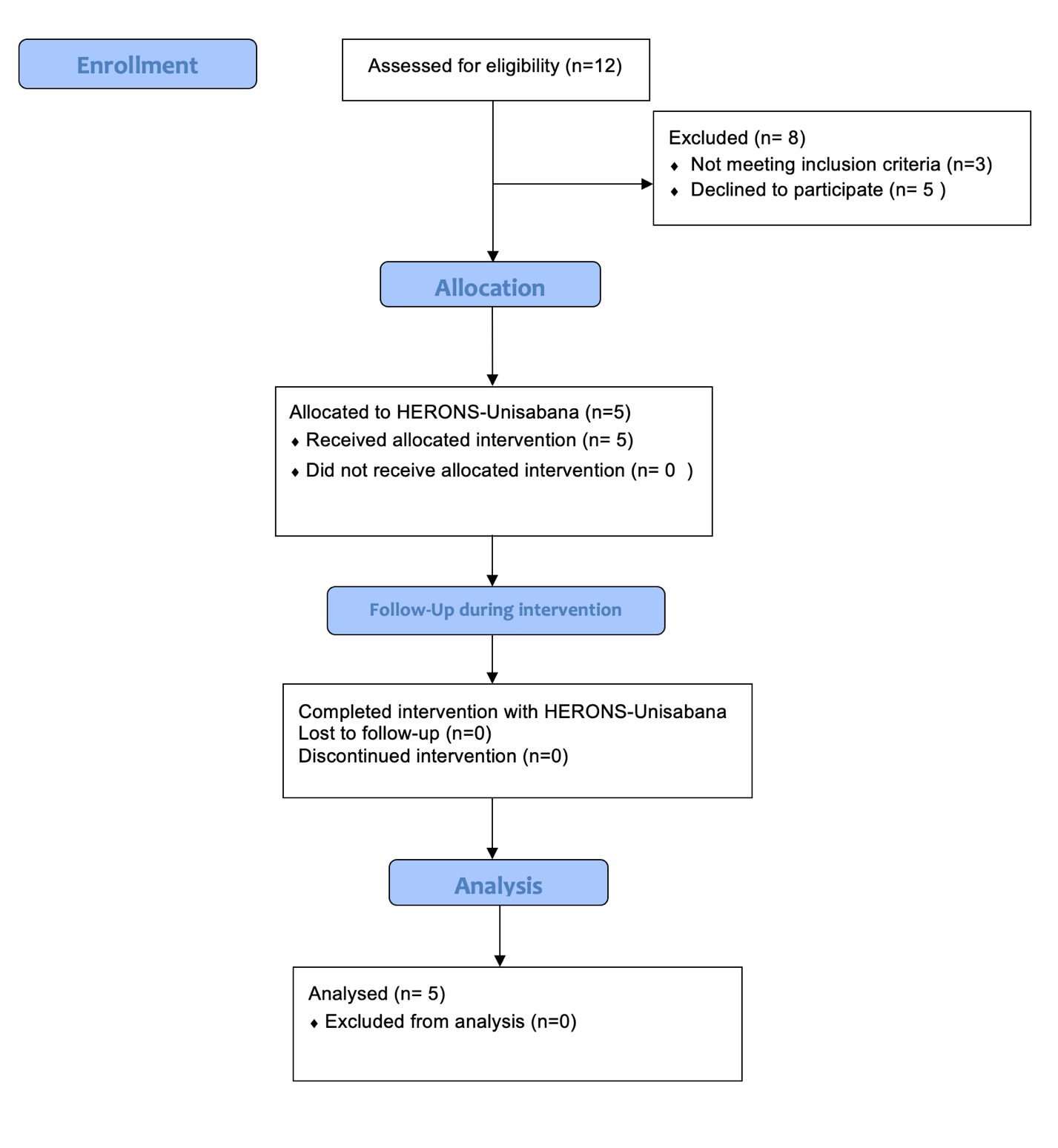


**REFERENCES**

[1] S. Bolsin, M. Colson, The use of the Cusum technique in the assessment of trainee competence in new procedures, Int J Qual Health Care 12(5) (2000) 433-8.

[2] S.V. Kemp, S.H. El Batrawy, R.N. Harrison, K. Skwarski, M. Munavvar, A. Rosell, K. Cusworth, P.L. Shah, Learning curves for endobronchial ultrasound using cusum analysis, Thorax 65(6) (2010) 534-8.

[3] N. Acute Respiratory Distress Syndrome, R.G. Brower, M.A. Matthay, A. Morris, D. Schoenfeld, B.T. Thompson, A. Wheeler, Ventilation with lower tidal volumes as compared with traditional tidal volumes for acute lung injury and the acute respiratory distress syndrome, N Engl J Med 342(18) (2000) 1301-8.
